# H5N1 Avian Influenza: A Narrative Review of Scientific Advances and Global Policy Challenges

**DOI:** 10.3390/v17070927

**Published:** 2025-06-29

**Authors:** Alison Simancas-Racines, Claudia Reytor-González, Melannie Toral, Daniel Simancas-Racines

**Affiliations:** 1 Universidad Técnica de Cotopaxi, Facultad de Ciencias Agropecuarias y Recursos Naturales, Carrera de Agropecuaria, Latacunga 050150, Ecuador; alison.simancas1000@utc.edu.ec; 2 Universidad UTE, Facultad de Ciencias de la Salud Eugenio Espejo, Centro de Investigación en Salud Pública y Epidemiología Clínica (CISPEC), Quito 170527, Ecuador; claudia.reytor@ute.edu.ec; 3 Universidad Espíritu Santo, Samborondón 090150, Ecuador; melannietoral@uees.edu.ec

**Keywords:** avian influenza H5N1, zoonotic transmission, One Health, pandemic preparedness, sustainable health systems

## Abstract

The H5N1 avian influenza virus continues to evolve into genetically diverse and highly pathogenic clades with increased potential for cross-species transmission. Recent scientific advances have included the development of next-generation vaccine platforms, promising antiviral compounds, and more sensitive diagnostic tools, alongside strengthened surveillance systems in both animals and humans. However, persistent structural challenges hinder global readiness. Vaccine production is heavily concentrated in high-income countries, limiting equitable access during potential pandemics. Economic and logistical barriers complicate the implementation of control strategies such as vaccination, culling, and compensation schemes. Gaps in international coordination, public communication, and standardization of protocols further exacerbate vulnerabilities. Although sustained human-to-human transmission has not been documented, the severity of confirmed infections and the rapid global spread among wildlife and domestic animals underscore the urgent need for robust preparedness. International organizations have called for comprehensive pandemic response plans, enhanced multisectoral collaboration, and investment in targeted research. Priorities include expanding surveillance to asymptomatic animal hosts, evaluating viral shedding and transmission routes, and developing strain-specific and universal vaccines. Strengthening global cooperation and public health infrastructure will be critical to mitigate the growing threat of H5N1 and reduce the risk of a future influenza pandemic.

## 1. Introduction

The highly pathogenic avian influenza (HPAI) H5N1 virus has undergone significant evolution since its initial detection in 1959 during an outbreak in domestic poultry in Scotland [1]. Although initially geographically restricted to Asia and primarily confined to avian populations, the virus has markedly expanded its range in recent decades. In 1997, the first documented human transmission occurred during an outbreak in Hong Kong, affecting 18 individuals and causing six fatalities, thereby signaling the zoonotic potential of the virus [1,2]. Since then, H5N1 has demonstrated remarkable persistence and geographic spread, reemerging in 2003 and becoming established in wild and domestic bird populations across Europe, Asia, and Africa [3]. In 2008, its presence further extended into multiple regions of the African continent [4], solidifying its status as a global health concern.

Since 2020, human infections with HPAI H5N1 have been reported in over 23 countries, with a case fatality rate approaching 50%, highlighting the severe clinical outcomes associated with these infections [5]. Although case reports have declined since 2022 [6], sporadic infections continue to be documented, primarily linked to direct contact with infected poultry or dairy cattle, and there remains no conclusive evidence of sustained human-to-human transmission [6]. While this situation provides temporary reassurance from an epidemiological perspective, it does not eliminate the risk of future viral adaptations enabling efficient interhuman transmission, as has been observed with other avian-origin influenza viruses.

Avian influenza A viruses, particularly the H5N1 subtype, pose a persistent threat, with pandemic potential due to their genetic recombination capacity, interspecies adaptability, and high lethality [3,7]. The continued circulation of these viruses in wild and domestic reservoirs, combined with the limited genomic surveillance and rapid response capabilities in many regions, amplifies global vulnerability [1]. From an economic standpoint, HPAI outbreaks have had a devastating impact on the global poultry industry. According to data from the World Organization for Animal Health (WOAH), 18,620 outbreaks in poultry were reported across 76 countries between 2005 and 2019 [8]. In 2020 and 2021 alone, more than 3000 additional outbreaks were recorded, resulting in the death or culling of approximately 15 million birds worldwide [9]. Between October 2021 and September 2022, 2520 HPAI H5N1 outbreaks in poultry were reported in Europe, and 3867 wild birds tested positive. Simultaneously, 131 infections in mammals—including bears, foxes, raccoons, skunks, and seals—were reported in the United States between May 2022 and February 2023 [10]. These findings underscore the virus’s growing capacity to infect a broad spectrum of host species and persist across complex ecological systems.

Mutations identified in mammalian HPAI H5N1 infections during the current panzootic have raised considerable concern within the scientific community, particularly regarding their potential role in facilitating viral adaptation to human hosts [11,12]. Avian influenza viruses (AIVs) remain a critical challenge for both public and veterinary health due to their widespread dissemination and, in certain subtypes, alarming case fatality rates [5]. Notably, subtypes carrying the hemagglutinin (HA) gene from the H5 and H7 lineages have accounted for at least 2634 laboratory-confirmed human cases worldwide, with more than 1000 reported deaths, emphasizing their high zoonotic potential and substantial lethality [5]. The genetic diversity of HPAI viruses, alongside their concurrent presence in multiple species, significantly complicates control efforts and calls for comprehensive, coordinated responses.

In this context, the aim of this review is to provide a comprehensive analysis of recent scientific advances related to the molecular biology, genetic evolution, ecology, and epidemiology of the HPAI H5N1 virus, as well as to examine the major global policy challenges associated with its prevention and control. The review further explores evidence-based mitigation strategies, including improvements in farm biosecurity, vaccination, zoning, and compartmentalization, with particular attention to regions where clade 2.3.4.4b viruses are established in poultry or where the risk of viral introduction via wild birds is elevated [13]. This work advocates for a multidisciplinary and intersectoral approach that integrates updated scientific knowledge with strengthened global health governance, acknowledging the limitations of relying solely on culling strategies to contain a pathogen with demonstrated pandemic potential.

## 2. Biological and Epidemiological Characteristics of H5N1

### 2.1. Genetic Evolution and Viral Adaptability

The highly pathogenic HPAI H5N1 virus belongs to the Orthomyxoviridae family and is characterized by a negative-sense, single-stranded segmented RNA genome encapsulated in pleomorphic particles [14]. The genome is composed of eight RNA segments—HA, neuraminidase (NA), matrix, nucleoprotein, polymerase basic 1, polymerase basic 2, polymerase acidic, and nonstructural 1—that collectively encode proteins essential for viral structure, replication, host interaction, and immune evasion [15,16].

AIVs are classified into genetic and antigenic subtypes based on combinations of their surface glycoproteins HA and NA. To date, 19 HA subtypes (H1–H19) and 11 NA subtypes (N1–N11) have been identified [16]. Genetic reassortment among these segments during co-infection events can give rise to novel viral subtypes such as H5N1, which exhibit varying degrees of transmissibility, virulence, and host adaptation [17,18,19].

AIVs are also categorized based on pathogenicity into low pathogenic avian influenza (LPAI) and HPAI viruses [20]. A key molecular determinant of pathogenicity lies in the cleavage site of the HA protein [21]. LPAI viruses typically possess a monobasic cleavage site (PEKQTR/GLF), recognized only by trypsin-like proteases present in limited tissues, restricting viral replication primarily to the respiratory and gastrointestinal tracts [22,23]. In contrast, HPAI strains exhibit a multibasic cleavage site (PQRESRRKK/GLF), enabling activation by furin-like proteases found ubiquitously in host tissues, thereby facilitating systemic viral replication across multiple organs [24].

Phylogenetically, AIV genomes are organized into hierarchical taxonomic levels: clades (e.g., 1.1, 2.2, 2.3), subclades (e.g., 2.3.2.1c, 2.3.4.4b), lineages (e.g., Eurasian, American), and genotypes (e.g., A1, B1, B3.13, D1.1). This classification framework supports genomic surveillance, tracing viral evolution, and development of targeted intervention strategies [21,22].

The evolutionary dynamics of H5N1 are shaped by antigenic drift and reassortment. Antigenic drift involves the accumulation of point mutations in HA and NA genes, potentially altering antigenicity and allowing escape from host immune responses [25,26]. Reassortment occurs when H5N1 co-infects a host alongside another influenza strain, resulting in progeny viruses with mixed gene segments. Such reassorted viruses can possess enhanced virulence or transmissibility [27].

A particularly concerning evolutionary pathway involves mutations in the HA receptor-binding domain, which increase affinity for human-like α2,6-linked sialic acid receptors in the upper respiratory tract, thus enhancing zoonotic and pandemic potential [28,29].

**Figure 1:**
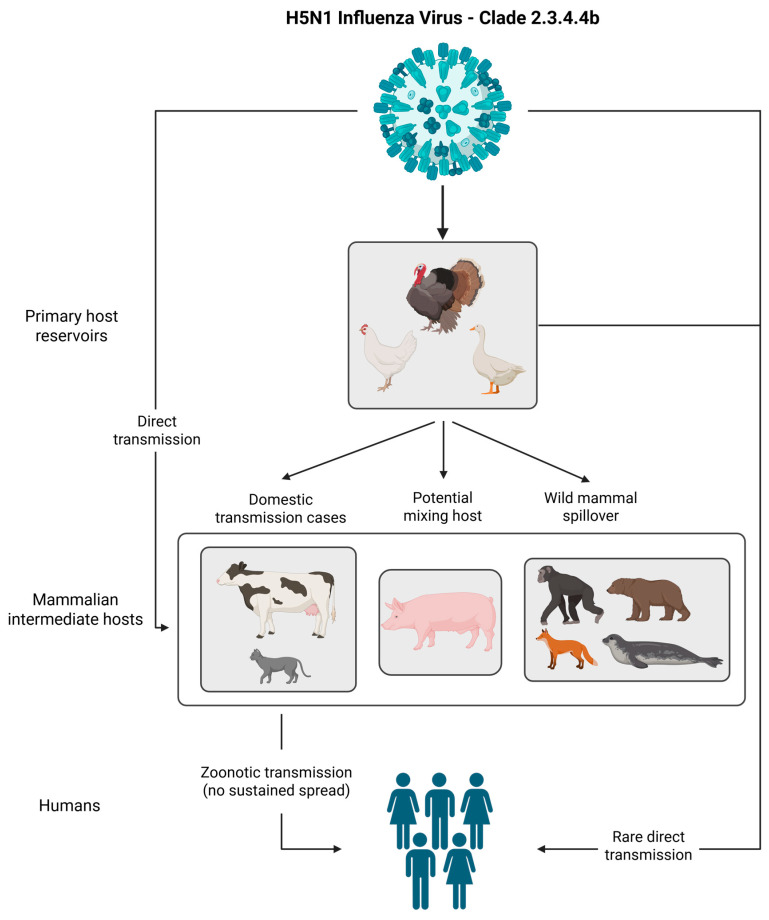
Zoonotic transmission and host adaptation pathways of H5N1 clade 2.3.4.4b. The virus primarily circulates in wild and domestic birds, with direct transmission to several mammalian species, including cattle, cats, and wildlife. Pigs are considered potential mixing hosts. Spillover events into wild mammals such as foxes, seals, and bears have been documented. Confirmed zoonotic transmission to humans has occurred, but there is no evidence of sustained human-to-human spread [25,27,30,33]. Created in https://BioRender.com.

Clade 2.3.4.4b has emerged as the dominant global lineage. This clade exhibits extraordinary genomic plasticity and adaptability, with genotypes such as B3.13 and D1.1 infecting a broad spectrum of avian and mammalian species (Figure 1) [30]. In 2020, reassortment events between clade 2.3.4.4b H5N8 and LPAI viruses in Europe and Central Asia led to the emergence of new genotypes AB and BB, combining polymerase complexes and surface proteins from distinct lineages [31,32,33]. As the virus spread to the Americas, further reassortment with local LPAI viruses produced genotypes such as B3.2 and B3.13, which have demonstrated the ability to infect marine mammals and dairy cattle (Table 1) [25,27,33].

### 2.2. Pathogenesis and Clinical Manifestations of H5N1 Infection in Humans

To date, human infections caused by clade 2.3.4.4b have generally resulted in mild disease, with only about 7% progressing to severe illness [35]. However, this same viral clade has been associated with lethal infections in various mammalian species [36].

The infectivity and pathogenicity of avian influenza viruses are influenced by their preference binding to specific sialic acid receptors on host cells. Theses viruses’ hemagglutinins exhibit a strong affinity for α-2,3-linked sialic acid receptors [35]. In humans, these receptors are predominantly found in the lower respiratory tract, including terminal bronchioles and alveoli, which may help explain the frequent occurrence of severe pneumonia in human avian influenza cases [37].

Respiratory symptoms are mainly caused by viral infection and cytopathic effects on the respiratory epithelium, while systemic manifestations such as fever and myalgia are linked to the release of pro-inflammatory cytokines like IL-6, TNF-α, and interferons [38]. Dysregulated innate immune responses and altered procoagulant activity have also been associated with rare, but severe complications such as acute necrotizing encephalopathy and an increased risk of fatal cardiovascular events [39].

Severe disease caused by the H5N1 virus in humans may arise through multiple mechanisms, either independently or in combination. These include [39]:

- Systemic viral dissemination beyond the respiratory tract (unlike seasonal influenza).
- Higher and prolonged viral replication resulting in direct cytolytic damage.
- Differences in tissue tropism of H5N1 viruses compared to seasonal strains.
- Exaggerated host immune responses triggered by H5N1 infection.

Although H5N1 has demonstrated the ability to spread beyond the respiratory system, being occasionally isolated from feces [40], serum [41], and in rare instances the central nervous system [42], immunohistochemical analyses at the time of the death frequently reveal only limited or no detectable viral presence in lung tissues, with few infected cells observed [43,44,45,46].

Avian influenza infection can result in severe complications, including respiratory failure and multiorgan dysfunction [47]. Respiratory manifestations are particularly critical, with primary viral pneumonia, acute respiratory distress syndrome (ARDS), and secondary bacterial pneumonia being the most concerning. ARDS, marked by diffuse alveolar damage and profound hypoxemia, stands out as a leading cause of mortality [37].

Severe cases may also involve cardiovascular complications such as myocarditis and septic shock [48], frequently progressing to multiple organ dysfunction syndrome (MODS), with simultaneous failure of the respiratory, cardiovascular, renal and hepatic system [49].

### 2.3. Pathogenesis and Clinical Manifestations of H5N1 Infection in Animals

Since 2020, numerous countries have reported outbreaks of the highly pathogenic avian influenza (HPAI) H5N1 virus belonging to clade 2.3.4.4b, resulting in devasting economic losses [50,51]. In domestic poultry, infection with HPAI H5 viruses typically leads to near 100% mortality rates [51]. In contrast, wild birds are naturally more resistant to HPAI viruses, often exhibiting mild or no clinical symptoms following infection [52,53]. However, contemporary H5N1 viruses from clade 2.3.4.4b have caused unprecedented mortality in wild bird populations [50,54].

There are several ways in which HPAI viruses can infect poultry flocks [55]. Pathogenesis begins when infectious virions are inhaled or ingested as trypsin-like enzymes in epithelial cells of the respiratory and intestinal tracts cleave the HA surface protein, facilitating multiple replication cycles in these tissues and releasing new virions [56,57].

Hemagglutinin (HA) binds to sialic acid receptors on host cell surfaces, enabling viral entry, while neuraminidase (NA) aids in the spread of infection [58]. Host restriction is largely determined by the specific linkage of sialic acid and galactose on the cell surface sialyloligosaccharides. Avian intestinal epithelial cells predominantly express NeuAc α2,3Gal linkages [59,60].

HPAI infection triggers a hyperinflammatory response know as a cytokine storm, which is strongly associated with severe lung injury in both avian and mammalian hosts [61]. Recent mechanistic studies have shown that H5N1 infection disrupts the alveolar epithelial barrier by targeting intercellular junction proteins through a process known as Itch-mediated proteasomal degradation [62]. Likewise, severe lung damage in chickens caused by clade 2.3.4.4b HPAI H5N1 has been linked to complex interactions between inflammatory macrophages, proinflammatory cytokines, and various cell types [63].

Differences in infection susceptibility and disease severity among avian species complicate surveillance and detection of emerging HPAI viruses, new viruses may circulate unnoticed, particularly in domestic ducks, which often act as asymptomatic carriers and can spread the virus over long distances, especially when not included in passive monitoring programs of dead birds. Wild bird species and individual birds play varying roles in the epidemiology of HPAI H5 viruses [64].

### 2.4. Transmission Dynamics and Zoonotic Potential

In avian hosts, H5N1 is transmitted primarily via contact with infected birds, their secretions (saliva, nasal discharge, feces), contaminated fomites (water, feed, surfaces), and predation or scavenging of infected carcasses [2]. The virus can survive in water and organic material for extended periods, particularly in cool and humid conditions, enhancing its environmental persistence and facilitating indirect transmission.

Human infection typically results from direct contact with infected poultry or contaminated environments. The highest risk occurs among individuals involved in poultry farming, processing, or culling activities, especially in settings lacking adequate biosecurity measures [26,65]. Infection can occur through inhalation of aerosols generated during slaughtering or defeathering, mucosal contact with contaminated hands or surfaces, and potentially through exposure to virus-laden dust. Experimental studies have confirmed fomite-mediated transmission [66].

Notably, H5N1 viruses that acquire specific mutations—particularly in HA or polymerase genes—have demonstrated the capacity for respiratory droplet transmission between mammals such as ferrets and guinea pigs [67,68]. These findings suggest that minor genetic changes could enable the virus to achieve efficient human-to-human transmission.

Although H5N1 remains poorly adapted to humans, recent zoonotic spillover events have intensified concern. In 2024, two human cases were reported—a fatal infection in Vietnam and a non-fatal case in Texas—both associated with contact with infected animals [25]. More importantly, the detection of H5N1 in dairy cattle in the United States represents an unprecedented epizootic event and raises questions about novel transmission routes, including potential food-chain exposure and secondary zoonoses [69,70,71,72].

In addition to sporadic human infections, H5N1 has been documented in various mammalian species, including minks (Spain), sea lions (Peru), seals (USA), foxes (Finland), and domestic cats (Poland, South Korea), indicating an expanding ecological range and capacity to infect phylogenetically diverse hosts [11,12,71,73,74,75,76,77,78,79]. While sustained mammal-to-mammal transmission has been observed in limited instances (e.g., mink farms), most mammalian cases appear to be isolated spillover events. Nonetheless, the increasing frequency of such infections suggests ongoing viral adaptation.

### 2.5. Virulence, Case Fatality, and Global Spread

H5N1 is one of the most virulent influenza viruses affecting humans. Since its initial detection in aquatic birds in China in 1996, the virus has caused severe disease characterized by rapid-onset pneumonia, acute respiratory distress syndrome, and multi-organ failure [25]. High viral replication in the lower respiratory tract and an exaggerated pro-inflammatory cytokine response (“cytokine storm”) contribute to high mortality rates.

According to World Health Organization (WHO) data, between 2003 and 2024, there were 963 laboratory-confirmed human cases of H5N1 infection, resulting in 465 deaths, yielding a case fatality rate of approximately 49% across more than 24 countries [5,80,81]. Most cases occurred in Southeast Asia, Egypt, and Africa, often in rural settings with close contact between humans and poultry. The persistently high case fatality rate underscores the virus’s pathogenic potential, even in the absence of sustained human-to-human transmission.

The ongoing HPAI panzootic, particularly involving clade 2.3.4.4b, has expanded H5N1’s geographic and host range. Between October 2024 and February 2025, 121 outbreaks were reported in poultry and 166 in wild birds and mammals across Africa, the Americas, Asia, Europe, and Oceania [82]. Initial spread occurred through migratory wild birds, which facilitated long-distance dissemination of novel genotypes. The virus has since become endemic in multiple wildlife reservoirs and has increasingly affected farmed and companion animals [33,70].

The scale and diversity of recent outbreaks are unprecedented. Mortality events have been documented in sea lions in Peru, seals in the United States, and minks in Spain [12,74,75,76]. Captive and domestic species—including foxes (Finland) and cats (Poland, South Korea)—have also been affected [77,78,79]. The widespread detection of H5N1 in dairy cattle marks a new phase in the virus’s evolutionary trajectory and raises concerns about interspecies transmission risks.

Although sustained transmission among humans has not yet occurred, the convergence of high virulence, expanding host range, and increasing environmental stability poses a serious threat to public health. Understanding the biological and epidemiological landscape of H5N1 is essential for anticipating and addressing the global policy challenges explored in the next section.

## 3. Scientific Advances in the Control and Prevention of H5N1

### 3.1. Vaccination Strategies and Immunization Efforts

Vaccination against HPAI may be implemented as an emergency or preventive strategy, provided it is part of an official immunization plan and carried out under the supervision of a competent health authority [83].

There are three main vaccine production methods against influenza: egg-based, cell-based, and recombinant vaccines [84]. The most common are egg-based vaccines, which use fertilized chicken eggs as the substrate for cultivating influenza viruses [85]. Within this category are inactivated vaccines, which contain virus inactivated by chemical agents like formaldehyde or beta-propiolactone, and live attenuated vaccines, which contain weakened viruses capable of inducing an immune response without causing disease [85].

Among the available vaccines for HPAI are inactivated vaccines, which may be monovalent [86] when they contain H5 or H7 strains or bivalent [87] if both subtypes are included. Monovalent and bivalent formulations may incorporate an NA that matches or differs from that of the circulating strain, depending on composition and prevailing virus [88].

There are also combination vaccines, which include antigens in addition to those from AIV. These are particularly useful for long-term immunization programs, as they reduce the number of required applications [89].

It is important to note that no vaccine guarantees optimal protection, even when antigenically matched, as vaccine efficacy can be influenced by host factors and viral evolution [14]. Specially, commercial inactivated vaccines currently do not guarantee optimal protection against H5N1 variants due to antigenic mismatches between circulating and vaccine strains [88]. Moreover, these vaccines rely on embryonated chicken eggs (ECEs), a method with limitations primarily due to limited ECE availability during emergencies [88,90].

Live recombinant vaccines, such as those based on fowl pox virus expressing the H5 protein, have shown effectiveness in chickens when administered on the first day of life [91]. However, their performance may be limited by the presence of high levels of maternally derived antibodies (MDAs) in neonatal chicks, as well as by preexisting immunity to the viral vector, particularly when common poultry pathogens are used as backbones [92,93].

In humans, vaccination remains the most effective strategy for preventing and controlling influenza, although its efficacy varies depending on the match between vaccine and circulating strains [14]. It continues to play a fundamental role in reducing morbidity, mortality, and the burden on healthcare systems.

#### 3.1.1. Development of Human Vaccines: Current Progress

Recent advances in AIV vaccine research have highlighted mRNA-based vaccines, which have shown strong potential in preclinical and early-phase trials. Several early-phase clinical trials are currently assessing the safety and efficacy of different mRNA vaccines in humans. These include vaccines developed by Moderna and Pfizer (in phase III trials), as well as those by Sanofi-Translate Bio and GlaxoSmithKline (GSK)-CureVac (in phase I) [94,95,96,97].

Since 2007, the United States has maintained a national stockpile of H5N1 avian influenza vaccine for active immunization of individuals aged 18 to 64 who are at higher risk of exposure, manufactured by Sanofi Pasteur Inc. [98,99]. AUDENZ, an inactivated vaccine indicated for the prevention of illness caused by influenza A H5N1 virus and manufactured by Seqirus, Inc., was approved by the FDA in February 2020 and added to the national stockpile [100,101]. Additionally, a vaccine developed by ID Biomedical Corporation of Quebec was approved by the FDA in November 2013 for individuals aged 6 months and older, also held in reserve for potential pandemic use and not commercially distributed [102,103].

However, it is still unknown whether they can induce neutralizing antibody responses against clade 2.3.4.4b strains currently circulating [104]. Seasonal influenza vaccines are not designed to provide protection against AIV type A [105,106].

Significant research efforts are underway to develop a universal influenza vaccine, targeting conserved viral epitopes to overcome the challenges posed by influenza virus genetic variability [107]. The goal is to induce broad cross-neutralizing immunity that offers protection against diverse viral strains [108]. Promising vaccine platforms include nucleic acid-based, protein-based, virus-like particles (VLPs) and live attenuated virus vaccines [109]. Currently, VLP and mRNA–DNA vaccines are at the forefront of influenza vaccine research [110], particularly in the context of AIV [14].

#### 3.1.2. Development of Animal Vaccines: Current Progress

In the poultry industry, efforts to develop H5 vaccines began promptly following the detection of the first highly pathogenic H5N1 virus in Guangdong in 1996 [5].

Vaccine technologies have historically encountered significant technical challenges related to formulation, administration, and the longevity of protective immunity [111]. Conventional first-generation vaccines have traditionally been developed through attenuation or inactivation of the infectious agent, aiming to induce protective immunity against disease [112,113,114].

Inactivated and live attenuated vaccines have been widely used. However, inactivated vaccines often exhibit low immunogenicity and limited ability to stimulate T cell-mediated adaptive immune responses, particularly against highly antigenically diverse pathogens. The potential instability of live attenuated vaccines can also pose challenges to achieving optimal vaccine efficacy [113].

Some of these limitations have been addressed by adding natural or synthetic adjuvants to inactivated vaccines to enhance their immunogenicity and by lyophilizing live attenuated vaccines to improve their stability. Nevertheless, despite these advancements, certain conventional vaccines still require booster doses to confer long-lasting immunity [111]. Several studies are also investigating the development of micro-and nanoparticles that encapsulate the vaccine and are engineered to enable a gradual release of antigens, thereby promoting sustained immune responses [115,116].

On the other hand, second-generation vaccines include, among others, those based on live recombinant viruses. The production of recombinant proteins typically involves sequencing the pathogen’s genome, identifying the genes that encode relevant antigens, and inserting those genes into expression systems such as mammalian cells, yeast, or bacteria. Once expressed, the proteins are isolated and purified for subsequent use as vaccines [111].

Third-generation vaccines have recently entered development, driven by advances in molecular biology that allow for rapid and cost-effective sequencing of viral genomes. In this approach, genetically modifiable viruses capable of expressing antigens from other pathogens serve as vectors for the production of recombinant vaccines [92].

Currently, commercially approved bivalent or trivalent vaccines are available for use in chickens (either in ovo or at day-old), utilizing various viral species that have been evaluated as recombinant vectors for AIV vaccines, including Newcastle disease virus (NDV), turkey herpesvirus (HVT) [117], fowl pox virus [118], adenovirus [119], infectious laryngotracheitis virus (ILT) [120], and Marek’s disease virus (MDV) [121], to express antigens along with an inserted H5N1 gene [122,123]. Newcastle disease virus (NDV)-based vectors offer significant advantages as a platform for bivalent vaccines targeting both NDV and highly pathogenic avian influenza (HPAI). Live attenuated NDV vaccines have proven to be safe and effective under field conditions. H5 and H7 vaccines using NDV as a vector have already been tested in practice. Recently, heterologous vaccination of chickens was used in production settings. This innovative strategy also helps overcome the limitations posed by the presence of maternal antibodies [124].

DNA vaccines are still under investigation in early stages of research. They are developed from a genetically modified version of the gene encoding the H5 hemagglutinin protein, designed to enhance recognition by the chicken’s immune system and elicit and effective defensive response [125]. The gene encoding this protein is inserted into a plasmid (a DNA vector), which is then administered to the birds. This enables their own cells to synthesize the protein and initiate a protective immune response against the virus [126].

These vaccines present several advantages over first- and second-generation vaccines, including simpler and faster production, a higher safety profile, and no requirement for high-level biosafety laboratories [111].

Virus-like particle (VLP) vaccines are a promising alternative against avian influenza in poultry. These particles mimic the structure of the virus without carrying genetic material or internal proteins, reducing risks such as recombination with wild-type strains and enabling DIVA strategies. They are produced using various expression systems and typically include viral proteins like HA, NA, and M1 to trigger and effective immune response [127,128].

Recently, VPL-based vaccines for avian influenza virus in poultry have been developed using various production platforms, including insect cells, with baculovirus, transfected mammalian cell lines, and plant-based systems [129,130,131,132,133].

Immune protection against avian viruses is primarily mediated by humoral and adaptative cellular immunity [134]. The level of neutralizing antibodies is routinely measured to assess immune protection in birds following infection or vaccination [135]. Thus, avian T-cell immunity plays a significant protective role against avian infections, and T cell-based vaccines (second- and third-generation vaccines) represent a major new advancement in global efforts to combat viral infections [136].

However, conventional vaccines aimed at inducing neutralizing antibodies often require regular administration and may become less effective over time due to immune pressure, which drives the continuous mutation of viral surface proteins in the circulating strains each year [136,137].

Despite this, it is important to note that implementing vaccination programs in poultry production can significantly reduce the economic losses associated with mass culling of flocks, thereby enhancing food security for the human population [123].

#### 3.1.3. Challenges in Production, Distribution, and Global Accessibility

The emergence of clade 2.3.4.4b HPAI H5N1 and its ability to spread from dairy cattle to other animals and humans pose a major threat to global public health. Developing effective vaccines and treatments has become an urgent priority to contain transmission and reduce the associated health and economic impact [104].

Despite the availability of high-yield influenza vaccines, large-scale production remains the biggest challenge in the event of a pandemic. Current global production capacity is around 1.2 billion trivalent doses, equivalent to 3.6 billion monovalent pandemic doses (15 µg) or approximately 1.8 billion two-dose treatments. However, over 85% of this capacity is concentrated in just seven manufacturers, while only 2% is based in low- and middle-income countries, which represent 38% of the global population. This disparity reflects a serious equity issue, as over 95% of annual doses are used in high- and upper middle-income countries [95].

If an avian influenza vaccine proves to be less immunogenic, it could require a higher antigen dose (e.g., 90 µg per dose), reducing total capacity to just 300 million doses. However, using adjuvants that allow dose-sparing to 7.5 µg per dose could raise production capacity back to 3.6 billion doses [95], significantly improving global access in a pandemic scenario.

### 3.2. Surveillance Systems and Early Detection

#### 3.2.1. Molecular and Serological Diagnostic Techniques

Virus isolation and identification remain the most reliable and widely accepted methods for confirming the presence of avian influenza virus (AIV) [138,139]. These methods allow for accurate identification of epidemic AIV subtypes, providing critical information for epidemiological surveillance and outbreak control [138,140].

The traditional method involves culturing the virus in embryonated eggs free of specific pathogens, followed by viral isolation [138,141]. However, this process is time-consuming and requires extended incubation periods [142]. Moreover, molecular diagnostics require that specimens be stored at 2–8 °C, and although biosafety level 3 laboratories are recommended for H5N1 isolation [20], practices may vary depending on national infrastructure and available resources.

Several enzyme-linked immunosorbent assays (ELISAs) have been developed for detecting AIV antibodies in poultry and other species, including humans [143]. Most ELISA methods rely on indirect detection through anti-immunoglobulin conjugates, enabling identification of immune responses to the virus [138].

Effective ELISA requires purified, concentrated live virus preparations as test antigens [144,145,146]. Notable innovations include competitive ELISA (CELISA) using recombinant baculovirus for AIV serology across multiple bird species, developed by Al Shafer et al. [147], and a double-antibody sandwich ELISA (DAS-ELISA) targeting the viral nucleoprotein, designed by Zhang et al., which improved detection sensitivity [147].

Additional ELISA variants have been explored to increase their epidemiological utility [147,148,149,150]. Overall, ELISA offers high sensitivity and specificity, but its low reproducibility remains a limitation for large-scale diagnostic deployment [151].

#### 3.2.2. Implementation of Early Warning Systems

Active surveillance in wild bird populations can be conducted through capture and sampling, facilitating virus detection in natural reservoirs [66]. During high-risk periods—such as migratory bird seasons—it is crucial to implement serological and virological monitoring. The use of sentinel birds, especially ducks due to their high susceptibility, within flocks enhances virus detection [64,66].

In poultry, syndromic surveillance has proven to be a reliable indicator of HPAI infection. Early signs include decreased feed and water intake, reduced egg production, and increased mortality, all of which can signal viral presence [64,152,153]. In wild birds, passive surveillance based on carcass analysis must be complemented by targeted sampling in strategic locations aligned with migratory bird routes [64].

Upon AIV detection in a market, farm, or country, all personnel in contact with birds must reinforce hygiene and biosecurity measures [154]. To prevent virus introduction (bio-exclusion), strict protocols should be enforced, including disinfection, access control, and staff management. If the virus is already present, biocontainment actions must be adopted to curb spreads such as isolating infected birds, restricting movement, and complying with sanitary regulations [64,66].

A standardized HPAI outbreak response protocol should include the following key procedures [155]:

- Ongoing clinical evaluation of affected birds and necropsies to identify characteristic signs.
- Immediate epidemiological analysis to identify transmission routes and contact networks.
- Collection of biological samples and submission to reference laboratories for confirmation.

In human cases, both the Pan American Health Organization (PAHO) and WHO emphasize strengthening surveillance of severe acute respiratory infections and influenza-like illness. Event-based surveillance is also recommended to detect outbreaks and enable timely intervention. This approach helps track virological, epidemiological, and clinical shifts in circulating influenza viruses that may impact human health [80,156,157].

Beyond active case finding, contact tracing in zoonotic investigations is essential. Raising clinical awareness and public communication about potential avian influenza infections is critical. It is also advised to reinforce surveillance near poultry farms, areas with previous human or animal cases, or suspected sources of infection to ensure rapid and effective response [80,156,157].

#### 3.2.3. The Role of International Surveillance Networks

The Food and Agriculture Organization (FAO), WHO, and WOAH encourage countries to prevent avian influenza at its source by implementing comprehensive surveillance programs covering both wild and domestic birds, including large commercial and backyard farms [80]. This approach aims to detect viral circulation across different environments and reduce the risk of cross-species transmission, including to humans.

These international agencies also recommend including H5N1 infection in the differential diagnosis of non-avian species, such as livestock and other farm animals, as well as captive populations at high risk of H5 exposure. This broader detection strategy facilitates early diagnosis and supports preventive measures to mitigate animal and human health risks [80].

### 3.3. Biosecurity Measures and Containment Strategies

Detailed records of poultry holdings near outbreak zones—including backyard flocks—are essential for monitoring and implementing effective control measures. Additionally, thorough epidemiological investigations are necessary to identify potential transmission routes and direct or indirect contacts with other farms, enabling the design of targeted containment strategies [20].

An epidemiological containment zone should be established according to the following criteria.

- Outbreak Zone: This includes poultry farms within a 1 km radius of the index case. In this area, drastic containment actions are recommended, such as depopulation and culling of affected flocks, to minimize viral spread and safeguard animal health [20].

Controlled depopulation of infected birds, combined with biosecurity protocols, movement restrictions, and active surveillance, is fundamental for reducing environmental contamination. Globally, HPAI containment approaches vary: Europe and North America primarily implement culling of infected or suspected birds, while China adopts a mixed strategy of culling and vaccination to reduce viral spread [5].

To ensure effective containment, poultry farms and facilities housing captive birds must implement immediate control measures, including destruction or decontamination of feed, equipment, and manure, to inactivate the virus and prevent new outbreaks [83].

- Observation and Surveillance Zone: Extending from 3 to 10 km beyond the outbreak center, this zone must follow strict containment protocols, such as prohibiting poultry transport and temporarily closing markets and shops selling live birds or eggs within the affected area [20].

#### 3.3.1. Trade Regulations and Restrictions on Live Poultry Transport

To limit disease transmission, authorities must immediately enforce movement restrictions on the affected farm and surrounding premises. A protection zone of at least 3 km and a surveillance zone extending up to 10 km from the outbreak epicenter should be designated [20,83,158].

If required, eradication measures may extend to neighboring farms or those with high-risk contacts with infected sites. This approach seeks to prevent further viral dissemination and reinforce epidemiological control across the impacted region [20,83].

#### 3.3.2. Economic and Food Security Implications

Since approximately 2020, H5N1 has been associated with massive and lethal outbreaks in wild and domestic birds across multiple continents, resulting in the death of millions of birds either from direct infection or targeted culling as a control measure [25].

Clade 2.3.4.4b viruses were first detected in North America in early 2022, introduced via migratory birds from Europe. They have since spread across the Americas, replicating the epidemiological impact observed in Eurasia and Africa. In North America, the virus has caused severe losses in the poultry industry and collapses in wild bird populations, threatening biodiversity and agroecological stability [159].

Between October 2024 and February 2025, approximately 11.4 million poultry deaths or cullings were reported in the Americas as part of control efforts against HPAI outbreaks. Notably, the number of poultry outbreaks reported in the first five months of the current seasonal wave already exceeds those recorded during the entire previous wave (October 2023 to September 2024), with 949 outbreaks in the current wave versus 786 in the previous one [82].

Likewise, the number of wild bird outbreaks reported in the same period is comparable to the total for the previous year, with 1028 outbreaks reported in the current wave compared to 1062 in the previous one. These figures highlight the ongoing spread of the virus and underscore the need to strengthen surveillance and sanitary control measures to curb its advance [82].

### 3.4. Antiviral Therapies and Human Treatment Options

#### 3.4.1. Current Antiviral Treatments (Oseltamivir, Zanamivir)

NA inhibitors such as oseltamivir (Tamiflu, oral) and zanamivir (Relenza, inhaled) have been widely used to treat influenza virus infections [160]. These antivirals exhibit normal inhibitory activity against NA, supporting their continued effectiveness in limiting viral replication [161]. For this reason, NA inhibitors (NAIs) remain the choice of treatment for influenza virus infections.

Nevertheless, continuous monitoring of viral sensitivity to these agents is essential. Surveillance through genetic sequencing and inhibition assays is needed to detect emerging resistance and preserve therapeutic efficacy [85].

Baloxavir marboxil (BXM), a cap-dependent endonuclease inhibitor, has shown efficacy in reducing replication of H5N1 and H7N9 subtypes [162]. According to a meta-analysis, BXM is safer and more effective than oseltamivir in influenza patients, supporting its use as a first-line treatment in confirmed influenza cases [162].

Chemoprophylaxis may be indicated for individuals meeting epidemiological criteria for H5N1 exposure [6].

#### 3.4.2. Emerging Research on Novel Therapeutic Approaches

New antiviral compounds are under investigation for both prophylactic and therapeutic use during influenza epidemics. These include polymerase inhibitors such as BXM and favipiravir, which have demonstrated efficacy in limiting viral replication and disease severity [163,164].

In vitro evaluations of baloxavir acid (BXA) in combination with other approved antivirals against different AIV subtypes have shown synergistic effects, positioning BXA as a strong candidate for combination therapies. BXA has consistently demonstrated equal or superior antiviral efficacy compared to oseltamivir across multiple studies [163,164].

Experimental antivirals like favipiravir have shown broad-spectrum activity against various AIV strains, making them promising options for future outbreak scenarios [37,163].

Additionally, proteolysis-targeting chimeras are under development. These agents use the ubiquitin-proteasome system to degrade pathogenic proteins, offering prolonged therapeutic effects and potentially reducing the likelihood of resistance compared to conventional inhibitors [7].

#### 3.4.3. Natural Medicine Against Influenza: The Role of Antioxidants and Immunomodulators

Oxidative stress triggered by RNA virus infections, such as influenza, plays a key role in disease pathogenesis. It contributes to processes like apoptosis, immune impairment, inflammation, and body weight loss [165]. Based on this, various natural compounds with antioxidant properties have been proposed as promising therapeutic alternatives.

Among these, Oximacro^®^, a patented cranberry (Vaccinium macrocarpon) extract, has demonstrated in vitro inhibition of both influenza A and B virus subtypes by blocking viral entry and attachment [166]. Likewise, CYSTUS052^®^, rich in complex polyphenols derived from Cistus incanus, showed potent antiviral activity against avian influenza H7N7 [167].

Echinaforce^®^ (EF), a standardized ethanol extract from Echinacea purpurea, contains powerful antiviral compounds. EF has been shown to block viral HA activity and prevent entry into host cells, even maintaining efficacy against Tamiflu^®^-resistant strains [168,169].

Other products, such as Ladania067^®^ (Ribes nigrum extract) [170,171], Mentofin^®^ (a water-soluble combination of peppermint and eucalyptus essential oils evaluated in poultry) [172], and black elderberry extract (Sambucus nigra) [173,174,175,176], have also shown antiviral activity, dose-dependent viral replication inhibition, and reduced cytopathic effects. These products often contain vitamins C and E, supporting their antioxidant action alternative remedies.

Additionally, traditional Chinese medicine (TCM), widely known for its plant-based therapies, has demonstrated synergistic effects when combined with conventional medicine [177]. A meta-analysis involving 30 studies and 3444 cases [178] confirmed that *ma-huang-tang* (a blend of Ephedra equisetina, Prunus armeniaca, Cinnamomum verum, and Glycyrrhiza uralensis) reduced fever either alone or when combined with neuraminidase inhibitors (NAIs) [179]. Andrographis paniculata was also reported to be safe and effective in reducing flu symptoms and shortening recovery time [180].

Randomized trails comparing oseltamivir with *maxingshigan-yinqiaosan* (a complex herbal formula) revealed similar clinical outcomes, including faster fever solution. TCM formulations are known to regulate key inflammatory signaling pathways like NF-κB and cytokines such as TNF-α and IL-6, essential for antiviral immune responses [181,182].

Furthermore, host-directed therapies such as monoclonal antibodies, immunomodulators, cell signaling inhibitors, and anti-inflammatory agents are being investigated as complementary treatments alongside direct-acting antivirals. These strategies are particularly useful in cases of drug resistance, severe disease, or immunocompromised patients [183,184,185,186,187].

The role of interferons (IFNs) in innate antiviral immunity is also well established. A phase II clinical pilot study in adult patients with influenza showed that the combination of IFN-α and oseltamivir was effective in resolving fever and other flu-related symptoms [188].

#### 3.4.4. Antiviral-Resistance Concerns

The high mutation rate of H5N1 enhances its ability to evade host immune responses and develop antiviral resistance, posing a challenge to current treatment strategies. Although specific H5N1 vaccines have been developed, their effectiveness is limited by the virus’s capacity for antigenic shift, which enables escape from vaccine-induced immunity. Moreover, the time required to adapt and distribute strain-specific vaccines can delay effective responses during emerging outbreaks [7].

The RNA genome of influenza A viruses lacks proofreading capability in its RNA-dependent RNA polymerase, leading to frequent mutations that may contribute to antiviral resistance and facilitate enhanced viral entry, replication, and pathogenesis in new hosts [73,189].

Two main classes of influenza antivirals exist: M2 protein inhibitors (amantadine and rimantadine) and NAIs (oseltamivir and zanamivir). However, H5N1 has demonstrated in vitro resistance to M2 inhibitors [85,190].

Resistance to NAIs has also been observed, particularly oseltamivir. Resistance is more common in children (4%–18%) and less frequent in adults (<1%). Oseltamivir resistance is typically linked to a point mutation (H274Y) in the NA gene that diminishes drug binding. In contrast, resistance to zanamivir is rarer due to the lower likelihood of relevant mutations affecting its mechanism of action [190].

The global response to H5N1 requires a multifaceted and coordinated approach that integrates scientific innovation, robust surveillance systems, strategic containment efforts, and equitable access to diagnostics, vaccines, and antiviral therapies. While important progress has been made in understanding the virus and developing tools to combat it, significant challenges remain, particularly regarding preparedness, production capacity, and resistance management. Strengthening international collaboration and investing in sustainable, inclusive public health infrastructure will be essential to mitigate the current threat and to better anticipate future outbreaks of this potentially pandemic virus.

## 4. Global Policy Challenges and Response to H5N1

### 4.1. International Cooperation and Governance Gaps

In May 2003, the Office International des Epizooties officially adopted the name WOAH. This entity is responsible for coordinating the global response to animal health emergencies, including zoonoses. It promotes animal health and welfare and aims to improve access to veterinary care worldwide [191]. Although avian influenza primarily affects animals, its outbreaks continue to pose significant public health risks. In response, the PAHO/WHO, in collaboration with the FAO and WOAH, urges member states to adopt collaborative and intersectoral approaches to ensure animal health and protect human populations [80,81,157,192,193].

The FAO, WHO, and WOAH emphasize the importance of working with local professional structures such as NGOs (e.g., VSF International), human health actors, and environmental organizations to effectively implement the One Health approach. This collaborative framework should focus on pooling expertise and building integrated strategies. These agencies call for continued advocacy efforts directed at Member States to anticipate the emergence and spread of communicable diseases across borders and at community levels, thereby improving preparedness and response capacity [194].

The WHO also works closely with the FAO, WOAH, and a network of laboratories to monitor the evolution of AIV, aiming to detect mutations that may increase risk to human health. Countries are encouraged to strengthen surveillance systems to detect human cases early, particularly those with limited prior experience in AIV surveillance given the virus’s expanding geographical range [157]. The European Centre for Disease Prevention and Control and the European Food Safety Authority have outlined actions grounded in the One Health approach. These include enhancing specific surveillance in both humans and animals, ensuring timely access to rapid diagnostics, and promoting intersectoral collaboration [195]. Preventive strategies such as poultry vaccination, especially among high-risk contacts, are also considered. Effective communication strengthened veterinary infrastructure, biosecurity implementation in farms, and reduced contact between wild and domestic animals are emphasized.

Despite these recommendations, significant disparities remain in national preparedness plans. For instance, while the European Union has coordinated simulation exercises and early warning systems, many low- and middle-income countries lack the infrastructure to implement such measures. Moreover, global efforts are often hindered by inconsistent reporting standards, limited interoperability of surveillance data, and insufficient funding for cross-sectoral outbreak preparedness. The lack of universally adopted contingency protocols results in fragmented responses, which delay containment and risk greater zoonotic spillover.

### 4.2. Economic and Trade Barriers

Integrating pandemic preparedness into national budgets, spending frameworks, and sectoral plans—preferably using domestic resources—is a key element in current global health policy discussions. Health emergencies have devastating economic impacts: future pandemics are projected to cause average annual losses equivalent to 0.7% of global gross domestic product [196]. This projection contrasts sharply with the relatively modest investment required for preparedness, which could yield high returns in economic savings, protected lives, and social stability [197].

A paradigm shift in the ecology and epidemiology of avian influenza has raised global concern as the disease spreads to new regions, causing mass mortalities in domestic and wild birds and a notable rise in mammalian cases, according to Dr. Gregorio Torres, Head of the Science Department at the WOAH [157]. HPAI has led to the death or culling of more than 633 million poultry worldwide between 2005 and 2024, peaking at 146 million birds affected in 2022. That year, 84 countries and territories reported outbreaks, highlighting the global scale and intensity of HPAI in recent years [192].

The WOAH recommends controlling the movement of susceptible domestic animals and their products and protecting individuals in close contact with them. Continuous monitoring of domestic and wild animal populations is essential, especially through investigation of unusual mortality events. Timely case reporting and the sharing of viral genetic sequences are critical to enhance understanding of HPAI epidemiology and support global response strategies [80,198]. To encourage early reporting, the WOAH advises national authorities to develop compensation plans for affected farm owners and producers. Such mechanisms promote cooperation, reduce the economic burden of control actions, and foster transparency and early response in the face of high-impact diseases [80].

### 4.3. Public Perception, Misinformation, and Risk Communication

Although poultry vaccination against HPAI may appear promising, it faces several limitations. One major issue is its impact on international trade: vaccinated poultry often face export restrictions unless specific conditions are met, affecting competitiveness [199]. Another key concern is the difficulty in distinguishing between infected and vaccinated animals—a problem known as differentiating infected from vaccinated animals (DIVA)—which complicates surveillance and trade regulations [199].

Additionally, vaccine efficacy varies due to viral mutations, and large-scale administration poses logistical challenges. Currently available vaccines must be injected, which is impractical in large poultry operations. Moreover, multiple doses may be needed for effective immunity. These challenges limit the feasibility of mass vaccination, particularly in outbreak settings or resource-limited countries [200]. Live attenuated vaccines offer advantages over inactivated ones, such as easier administration and stronger, longer-lasting immunity with fewer doses. However, they carry risks of environmental dissemination or reassortment with wild-type viruses, potentially leading to new variants [201].

Between 24 March 2024 and 17 January 2025, targeted H5 surveillance led to the monitoring of over 13,400 individuals exposed to infected animals. Of these, more than 600 were tested for possible human infection as part of zoonotic transmission prevention and control strategies [80]. The PAHO has launched an interactive dashboard for tracking H5N1 cases in the Americas. This tool improves access to real-time data on avian, mammalian, and human outbreaks, supporting public health and veterinary decision-making. The platform includes interactive maps and tables with data sourced from the WOAH. Through this initiative, the PAHO strengthens active surveillance and early-response capacities [202].

Beyond data sharing, effective communication must address public concerns and counter misinformation. Risk communication strategies should leverage trusted community figures, local languages, and culturally sensitive messaging. In addition, partnerships with social media platforms can help mitigate the spread of misinformation during outbreaks. Lessons from COVID-19 demonstrate that transparent messaging, early engagement with communities, and addressing misinformation in real time are critical to securing public compliance and building trust.

Given the substantial impact of HPAI outbreaks in recent years, it is crucial to implement short-term preparedness strategies and long-term prevention plans. These should focus on high-density poultry areas and vulnerable production systems to reduce outbreak risks and mitigate their health and economic consequences [203]. The emergence of a novel influenza strain has historically—and likely will continue to be—a potential pandemic threat. However, by learning from past outbreaks and applying scientific and technological innovation through multidisciplinary approaches, it is possible to enhance surveillance, improve preparedness, and respond more effectively to future crises [27].

Significant gaps remain in pandemic preparedness. For example, home or point-of-care tests currently cannot differentiate HA serotypes, limiting specific strain detection. Monoclonal antibodies show therapeutic potential, but remain cost-prohibitive for many low- and middle-income countries. Despite promising advances in mRNA and viral vector vaccine platforms, no formulations have yet received specific authorization for influenza [95].

### 4.4. Inequities in Resource Allocation

Addressing social inequalities is critical in managing HPAI. Control measures such as poultry trade restrictions or sanitary lockdowns can disproportionately affect vulnerable populations—small-scale producers, rural workers, and low-income communities—exacerbating preexisting disparities, as seen in previous pandemics [85,204]. It is also essential to understand the current challenges and limitations in human and animal vaccination against AIV, particularly in at-risk groups and regions with limited veterinary and public health services. This knowledge supports decision-making on booster doses and effective prophylactic strategies. Given H5N1’s potential to become endemic or pandemic, the development of equitable and sustainable interventions must remain a top public health priority [85,205].

Vaccine manufacturing is heavily concentrated in Europe and North America, a major limitation during a pandemic. The current trivalent vaccine production capacity—estimated at 300 million doses annually—would fall short of global needs. Furthermore, pandemic-specific strains require urgent antigen reformulation [110]. To increase coverage, monovalent pandemic vaccines are being considered, which would allow for three times more individual doses. Additionally, incorporating immunopotentiating adjuvants could enable intradermal delivery using up to tenfold-lower antigen doses while maintaining protective immunity [110].

Nevertheless, due to logistical and infrastructure constraints, no country is expected to have enough vaccine doses during the early months of a pandemic. Large-scale production is not expected to begin until three to six months after the pandemic strain is identified, highlighting the need for contingency planning and equitable access strategies [110].

Key stakeholders in emergency preparedness and health security include the human, animal, and environmental sectors. These three domains form the internationally recognized One Health approach, essential for effective multisectoral coordination. Its significance lies in recognizing the impact of zoonotic disease transmission on public health and the need for integrated, collaborative responses [197]. The PAHO/WHO recommends reinforcing intersectoral actions through standardized protocols that integrate all relevant sectors and clearly define roles to facilitate information exchange and analysis. A comprehensive One Health-based early detection and rapid response strategy should account for both human and animal health risks, improving overall prevention and control capabilities [80,197].

Within the One Health framework, expanding global zoonotic influenza surveillance systems in both animals and humans is vital. Epidemiological, virological, and pathogenesis research must also be enhanced to generate evidence-based risk assessments and timely pandemic alerts. In parallel, the development of universal influenza vaccines and new antivirals represents a promising strategy to improve protection and reduce future pandemic impacts on global public health [206].

## 5. Conclusions and Future Perspectives

The H5N1 virus has evolved into more diverse and virulent clades, such as 2.3.4.4b, with increasing zoonotic potential. Scientific advancements have included the development of new vaccine platforms, stockpiled prepandemic vaccines, and ongoing efforts toward a universal influenza vaccine. Novel antivirals like BXM and therapeutic combinations have demonstrated greater efficacy against emerging strains. Diagnostic techniques have improved through molecular and serological tools such as polymerase chain reaction, competitive ELISA, and sandwich ELISA. Surveillance systems for both animals and humans have been strengthened with multisectoral efforts under the One Health approach [80,192,193].

Nonetheless, significant structural challenges persist. Vaccine production remains concentrated in high-income countries, limiting equitable access in the event of a pandemic. Economic barriers hinder sustained implementation of control strategies—such as culling, biosecurity, and compensation—and there are persistent gaps in international cooperation, governance, and protocol standardization. Trade restrictions, the challenge of DIVA, and public distrust further complicate the implementation of vaccination campaigns [197].

The history of influenza pandemics, alongside the ongoing evolution of the HPAI H5N1 epizootic and the severity of associated human infections, stands as a stark warning of the very real risk of a new influenza pandemic. Conservative estimates suggest that such a pandemic could result in up to 350 million deaths globally, severely disrupting healthcare systems, societal structures, and economies. In light of this, the WHO has urged countries to prepare and implement comprehensive national pandemic preparedness plans that integrate both public health interventions and pharmaceutical strategies to mitigate impacts and safeguard populations [207].

Although sustained human-to-human transmission of H5N1 has not yet been documented, the severity of confirmed cases underscores the urgent need for ongoing surveillance and robust public health measures [208]. This concern is heightened by the significant global increase in HPAI H5N1 activity since 2021, which has caused high mortality rates in wild and domestic birds, along with sporadic mammalian infections, raising the risk of zoonotic spillover [209].

The FAO and WOAH recommend leveraging existing networks, such as animal health cooperation partnerships, to implement on-the-ground actions. These include supporting global disease eradication campaigns for high-priority illnesses like peste des petits ruminants, foot-and-mouth disease, and rabies, as well as promoting good livestock practices, especially biosecurity, to prevent the introduction and spread of infectious diseases. These networks must also play active roles in epidemiological surveillance and crisis response mechanisms [194].

Governments worldwide should invest in developing an H5N1-specific vaccine and advance phase I and II clinical trials as part of broader preparedness efforts for potential sustained human transmission [210]. In this context, the FAO, WHO, and WOAH have intensified efforts by convening international experts to monitor viral evolution, assess risks, and update guidance to contain viral spread. Simultaneously, they are working with countries to enhance emergency response and promote cross-sectoral and international collaboration [29].

To bridge current knowledge gaps, future research should expand beyond sick or deceased wild animals to include molecular and serological testing of asymptomatic mammalian species. Studies in both wild and domestic animals—particularly terrestrial and marine mammals—are essential to assess the true scope of virus transmission [211]. Investigations should also correlate clinical symptoms with pathological findings in infected animals over time, explore transmission routes in both animals and humans, evaluate the duration of viral shedding by species, and develop optimal testing protocols for ongoing surveillance. These efforts are vital for improving human infection risk assessments and formulating effective prevention and mitigation strategies [211].

## Figures and Tables

**Table 1:** Distribution of genotypes and host species affected by clade 2.3.4.4b.

Genotype	Region of Origin	Year	Affected Species	Key Notes	References
AB/BB	Europe, Central Asia	2020	Poultry, wild birds	Result of reassortment between H5N8 and LPAI viruses	[31,32,34]
B3.2	Americas	2021	Wild birds, marine mammals	Reassortment in the Western Hemisphere	[25,27]
B3.13	Americas	2022	Seals, minks, dairy cattle, domestic cats	Genotype with high interspecies adaptability	[25,27,33]
D1.1	Asia	2022	Birds, mammals (emerging data)	Involved in sporadic infections, monitoring underway	[30]

Abbreviations: LPAI: low pathogenic avian influenza.
